# A role of local VTA GABAergic neurons in mediating dopamine neuron response to nicotine

**DOI:** 10.1186/1471-2202-16-S1-P137

**Published:** 2015-12-18

**Authors:** Ekaterina Morozova, Maxym Myroshnychenko, Marie Rooy, Boris Gutkin, Christopher C Lapish, Alexey Kuznetsov

**Affiliations:** 1 Department of Physics, Indiana University, Bloomington, IN, 47405, USA; 2 Program in Neuroscience, Indiana University, Bloomington, IN, 47405, USA; 3 Group of Neural Theory, ENS, Paris, 75005, France; 4 National Research University Higher School of Economics, Moscow, 101000, Russia; 5 Addiction Neuroscience Program, IUPUI, Indianapolis, IN, 46202, USA; 6 Department of Mathematics, IUPUI, Indianapolis, IN, 46202, USA

## 

**Figure 1:**
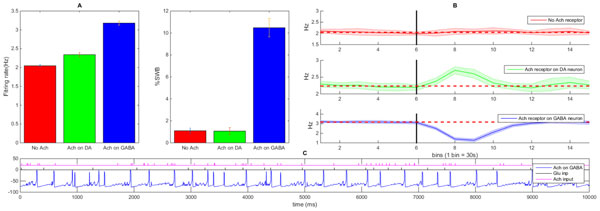
****A) **Quantification of Ach mediated firing of DA neuron (firing rate and %SWB) **B) **Nicotine-elicited modifications in firing rate of DA neuron**. Vertical black lines indicate nicotine injection. Horizontal dashed lines indicate the baseline firing rate. **C) **Example voltage trace of DA neuron (nAch receptor on GABA neuron only).

The local circuitry of the tegmental area (VTA) consists primarily of dopamine (DA) and GABA neurons. Interactions between DA and GABA neurons are critical for regulating DA neuron activity, and thus DA efflux throughout the brain. One striking example that demonstrates the significance of local interactions between DA and GABA neurons is related to nicotine reinforcement. Experimentally, it was shown that activation of nicotinic acetylcholine (nAch) receptors on GABA neurons by Ach leads to an increase in both firing and bursting of the DA neuron, while nicotine produces an opposite effect [1]. In order to investigate the mechanism of this GABA-mediated effect, we created a biologically plausible model of local VTA microcircuitry. The model network consists of a population of GABA neurons innervating one DA neuron. DA neuron dynamics are described by a conductance based model; which includes intrinsic and synaptic currents conducted by NMDA, AMPA, GABA, and nAch receptors. GABA neurons that are described by Wang-Buszaki equations provide inhibitory drive to the DA neuron. Excitatory inputs (Glu and Ach) to DA and GABA neurons are modeled as Poisson-distributed spike trains. Ach pulses act as synchronizing inputs to a population of GABA neurons, due to a transient activation of GABA nAch receptors, while nicotine persistently activates nAch receptor, causing an increase in firing frequency of GABA neurons. Modeling revealed that synchronization of GABA neurons by cholinergic input could provide a mechanism for the elevation of DA neuron firing frequency and bursting. Synchronized GABA inputs act via phasic disinhibition that promotes rebound spikes, but is conditioned on the presence of depolarizing currents to the DA neuron. The opposite effects of nicotine applied to the GABA neuron only (decrease in firing and bursting of DA neuron), could be the result of desynchronization in population of GABA neurons, produced by tonic activation of nAch receptor. A desynchronized GABA population provides constant inhibition to the DA neuron that suppresses firing (see Figure 1). These data highlight the important and powerful role local circuit dynamics VTA. Furthermore, synchrony amongst GABA neurons seems to be a critical intermediary that regulates DA neuron activity and, by extension, DA release throughout the brain.
